# Accessibility of healthcare in rural Zimbabwe: The perspective of nurses and healthcare users

**DOI:** 10.4102/phcfm.v12i1.2245

**Published:** 2020-05-14

**Authors:** Manenji Mangundu, Lizeth Roets, Elsie Janse van Rensberg

**Affiliations:** 1 Department of Health Studies, School of Social Sciences, University of South Africa, Pretoria, South Africa

**Keywords:** accessibility of healthcare, challenges, nurses, healthcare users, rural areas

## Abstract

**Background:**

Accessibility of healthcare in rural areas is globally impeded by physical, material, human, financial and managerial resources and societal barriers in the healthcare system. Developing countries like Zimbabwe are significantly affected.

**Aim:**

The aim of this article was to share the perspectives of nurses and healthcare users (HCUs) in the rural areas of Zimbabwe with regard to the accessibility of healthcare.

**Setting:**

The study was conducted at 45 rural health facilities in Chegutu district, Mashonaland West province and Masvingo district in Masvingo province, Zimbabwe.

**Methods:**

A self-administered questionnaire (for professional nurses) and a structured interview questionnaire (for HCUs) were utilised to gather data in a cross-sectional survey. Two districts were randomly sampled from 59 districts. All nurses working in 45 public health facilities in the selected two districts, who were willing and available to participate, were included. Ninety nurses participated in the study. The HCUs were selected through a multistage sampling technique. The sample size for HCUs was calculated by using Dobson’s formula, and 445 HCUs were included via convenience sampling.

**Results:**

Nurses reported challenges such as work overload because of staffing shortages (55%) and the supply of necessary medical drugs that lacked consistency in both the quantity and type ordered(46.7%). The challenges faced by HCUs included long distances from villages to health facilities (86%), unaffordability of transport costs and lack of access to medical drugs (59.95%), causing them to seek assistance from traditional healers (43%).

**Conclusion:**

Both the nurses and HCUs perceived grave challenges regarding access to health facilities, health workers and medical drugs, all of which are bound to have an impact on the health of communities in rural Zimbabwe.

## Background

Ensuring the well-being of citizens is the goal of all healthcare delivery systems,^1^ where individuals have the right to access good-quality healthcare. Allocating the relevant and required resources (e.g. financial resources) contributes to the accessibility of health facilities, essential medical drugs and competent and adequately trained health workers, as well as medical supplies.^2^

In developing countries like Zimbabwe, access to healthcare services is often influenced by long distances and travel times to health facilities, the availability of financial resources to travel or pay for care and the availability of medical drugs as well as competent healthcare workers.^3^ For example, in Zimbabwe, people in rural areas often have to walk between 10 km and 50 km to access the nearest health facility.^3^

Access can be further impeded by a lack of infrastructure, such as dirty roads that are not maintained, resulting in poor road conditions and potholes that create barriers to transport.^4^ In Zimbabwe, because of economic challenges, bridges that have collapsed because of rain are not repaired, hindering travelling of patients during critical times^5^ and negatively affecting the timely delivery of medical drugs and medical supplies to rural health centres.

Even where healthcare services are available and affordable, access to medical drugs is limited.^6,7^ There is often a shortage in the supply of medical drugs, especially in the rural parts of Zimbabwe. It is evident that the economic crisis in Zimbabwe has also led to a shortage of medical supplies and equipment in public health facilities, leaving professional nurses with limited options to provide treatment.^8^

The economic crises has caused community outreach programmes to be closed, as they were likely to place a further burden on the few nurses available.^9^ The family planning distribution programme has crumbled, as family planning drugs are not available, and thus the birth rate has increased.^10^ There were challenges with access to antiretroviral drugs for people living with HIV in the rural areas because of shortages, transportation challenges and nurses’ attitudes at designated rural health facilities.^11^

Zimbabwe has also failed to meet the minimum 15% annual health budget allocation as indicated in the Abuja Declaration of 2001, where African leaders agreed to allocate 15% of their countries’ total annual budget to the health sector.^12^ Although some efforts were made by Zimbabwe’s government to reverse the significant declines in the health allocation of the annual budget in 2010 (12.3% of the total allocated annual budget), the increase was reversed in subsequent years to 7% in 2012, 8.2% in 2013, 7.3% in 2015 and 8.3% in 2016.

Evidence of the dire need to investigate how nurses and healthcare users (HCUs) perceive accessibility to healthcare included the higher rates of vital statistics like infant mortality and maternal mortality rates (MMRs). Infant mortality also increased from 53 per 1000 in 1992 to 56.4 per 1000 in 2016.^13^ The MMR in Zimbabwe rose from 695 per 100 000 in 1999 to 960 per 100 000 in 2011 and fell in 2015 to 443 per 100 000, attributed to a rise in attendance by skilled health staff. Despite the fall in MMR, 443 per 100 000 is way above the target of 70 per 100 000 according to the Sustainable Development Goals (SDGs) (Goal 3).^13^

### Aim

The aim of this article was to share the perceptions of nurses and HCUs regarding accessibility to healthcare in rural Zimbabwe.

### Objective

The objective was to share and describe the challenges to accessing healthcare as perceived by (1) the nurses working in healthcare facilities in rural Zimbabwe and (2) the HCUs in rural Zimbabwe.

## Methods

### Study design

A cross-sectional quantitative survey was conducted in two rural districts of Zimbabwe.

### Population and sample

The setting of the study included 1170 public health facilities in 62 districts of Zimbabwe, of which 997 are in rural areas.^14,15^ Within the 62 districts, 11 054 nurses were working in rural areas,^16,17^ serving a population of 8 777 094 people.^18^

The researcher used impartial random sampling to select the districts. The names of all 62 districts, written on separate pieces of paper, were placed in a box. Two districts were selected randomly by a blindfolded individual. The selected districts had a total population of 350 757 people living in the rural areas^18^ and 45 rural public health facilities.

All nurses (120) working in the 45 public health facilities in the selected two districts, who were willing and available to participate during the time of data collection, were recruited. Ninety nurses agreed and participated in the study.

The households in the catchment areas of the 45 public health facilities were sampled by using a multistage sampling technique. The households were clustered in each catchment area, by using the distance to the health facilities, as follows: those within a ≤ 5-km radius and those within a > 5-km radius. In these clusters of ≤ 5-km and > 5-km radii within each catchment area, HCUs (five HCUs from a nearby area and five HCUs from a distant area) were conveniently sampled based on the availability and willingness of respondents aged ≤ 18 years to participate in the study. The sample size for HCUs was calculated by using Dobson’s formula for descriptive studies.^19^

### Research instrument

A self-administered questionnaire for the nurses and a structured interview questionnaire for the HCU respondents were developed. The questionnaires were developed based on a literature review on accessibility of healthcare. The questions were based on the inputs needed to enhance access to healthcare, as described within the Systems Model illustrated by Forester 2009 (Figure 3).^20^ The questionnaires were assessed for validity by the two supervisors and the statistician, as well as the appointed scientific review committee. The instruments had been pre-tested by purposely selected professional nurses (four nurses) at two health facilities and 20 HCUs in the catchment areas before the data collection commenced.

### Data collection and analysis

The self-administered questionnaire was distributed to the professional nurse respondents, and the structured interview questionnaire was administered by research assistants to the HCU respondents. The nurses completed the self-administered questionnaire and returned it in a sealed envelope, in a safe box in the office of the nurse-in-charge. After 2 weeks, the researcher followed up in person to collect the completed questionnaires at each health facility from the sealed box. During the study, 90 nurses completed these self-administered questionnaires.

Data were gathered from the HCU respondents by research assistants by using the structured interview questionnaire. The research assistants assisted respondents where needed with the completion of 445 questionnaires. This approach, as suggested by Johnson and Christensen,^21^ allowed the research assistants to elaborate and assist respondents in understanding the questions during data collection.

The data collected by using both the types of questionaires were separately entered in Census and Survey Processing System (CSPro) Version 4, and exported to Statistical Package for Social Sciences (SPSS) Version 22 for analysis. Descriptive statistics, showing frequencies and cross-tabulation, was used to present the decoded data.

### Ethical consideration

All the protocols for ethical consideration were followed, including obtaining ethical approval from the Scientific Research Ethics Committee at the Department of Health Studies of the University of South Africa (Reference Number: HSHDC 240/2013) received on 23 October 2013. Approval to conduct the research in Zimbabwe was obtained from the Medical Research Council of Zimbabwe on 03 July 2014 (Approval Number: MRCZ/A/1832).

### Findings

Forty-one out of 90 (45.55%; *f* = 41) professional nurses were between 26 and 35 years of age, and 39 out of 90 (43.33%; *f* = 39) were between 36 and 50 years. Only 3 out of 90 (3.33%; *f* = 3) professional nurse respondents fell in the age range of 18–25 years, 7 nurses with 51+ years, representing 7.77% (see Table 1). Of these 90 professional nurses, 21 (23.3%) were male and 69 (76.6%) female.

**TABLE 1:** Frequency distribution: Demographic characteristics of the study respondents.

Demographic characteristics	Professional nurse respondents *N* = 90		Healthcare user respondents *N* = 445	
	Frequency (*f*)	Percent	Frequency (*f*)	Percent
**Gender**				
Male	21	23.33	147	33.03
Female	69	76.66	298	66.96
**Age**				
18–25	3	3.33	73	16.40
26–35	41	45.55	102	22.92
36–50	39	43.33	121	27.19
51+	7	7.77	149	33.48
**Education level**				
Primary school	-	-	72	16.17
Secondary school	-	-	333	74.83
College certificate	60	66.66	12	2.69
College diploma	30	33.33	15	3.37
Degree	0	0	13	2.92
**Average monthly income**				
Less than US$100	-	-	271	60.89
US$100–250	-	-	128	28.76
US$251–500	57	63.33	34	7.64
US$501–750	32	35.55	7	1.57
US$751–1000	1	1.11	4	0.89
US$1000+	-	-	1	0.22
**Sources of income (*N* = 442)**				
Self-employment	-	-	224	50.67
Full-time employment	-	-	116	26.24
Part-time employment	-	-	35	7.91
Remittances	-	-	67	15.15

US, United States.

The research findings and/or perceptions of the 90 nurses and 445 HCUs are discussed according to the health systems model inputs needed for accessibility to healthcare, namely physical resources, material resources, human resources and financial resources.

#### Physical resources

**FIGURE 1:**
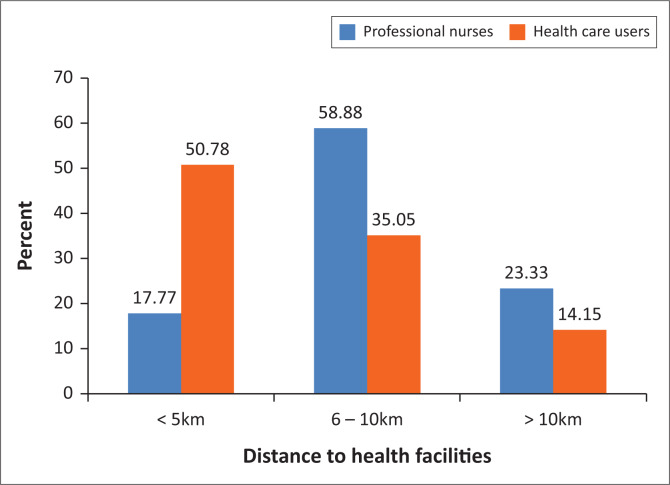
Distance to health facility (professional nurses: *N* = 90; healthcare users: *N* = 445).

HCUs indicated the walking distance to the health facilities in rural areas as a challenge, 35.05% HCUs walk between 6 km and 10 km (*n* = 445; *f* = 156) and 14.15% walk more than 10 km to the nearest health facility (*n* = 445; *f* = 63) (Figure 1). Because of this challenge, 44.94% of the HCUs (*n* = 445; *f* = 200) use the option of consulting traditional healers for healthcare, as they live within their villages and are more accessible.

Transport is known to bridge the problem of distance, but 64.74% of HCU respondents (*n* = 417; *f* = 270) reported that accessing public transport is a challenge. The non-availability of transport was identified as a challenge by both 64.74% HCUs (*n* = 417; *f* = 270) and 97.77% nurses (*n* = 90; *f* = 88). The situation was even more challenging because of the poor road conditions arising from a lack of maintenance. The inaccessibility of public transport and poor quality of the roads contribute to the fact that HCUs (*n* = 445; *f* = 266; 60.59%) have to walk to health facilities, and if they have to walk more than 10 km, they do not access healthcare and it becomes a severe challenge, with a negative impact on the health of patients. The unavailability of transport also affects the supply of material resources to health facilities.

#### Material resources

**FIGURE 2:**
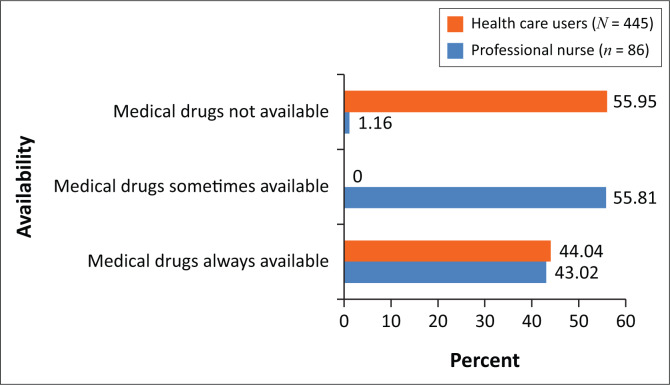
Medical drugs’ availability at the rural health facility (professional nurses *N* = 86 and healthcare users *N* = 445).

Medical drugs play an important role in saving lives, and nurses perceived the shortage of medicines as their greatest challenge when providing healthcare in rural areas. According to the nurses (*n* = 90; *f* = 87; 96.66%), medical drugs were supposed to be delivered on a quarterly basis, but deliveries lacked consistency in both the quantity and type (*n* = 90; *f* = 48; 46.7%) ordered. Similarly, HCUs viewed access to medical drugs as a challenge, with 59.95% of HCU respondents (*n* = 445; *f* = 249; 59.95%) reporting failure to obtain medicines during their last visits to health facilities in the rural areas (Figure 2). The nurses (*n* = 90; *f* = 68; 75.55%) reported that medical drugs for chronic conditions were rarely available and that this had a negative impact on the treatment of patients. The non-availability of medical drugs leads to a high risk of relapse and non-adherence by patients suffering from chronic diseases, which has a negative impact on the health outcomes in a community or country.^21^

Nurses (*n* = 90; *f* = 65; 72.22%) and HCUs (*n* = 311; *f* = 133; 42.76%) perceived the inaccessibility of medical supplies as a severe challenge, as patients are requested to bring their own medical supplies like bandages, cotton wool, intravenous fluids, candles and methylated spirits. The economic crisis results in HCUs experiencing financial constraints, rendering them unable to bring their own medical supplies, resulting in poor health outcomes.

#### Human resources

One of the key factors of accessibility to healthcare services is ensuring that people living in rural areas have access to trained health workers, but 60% of the nurses (*n* = 86; *f* = 54) and 44.94% HCUs (*n* = 445; *f* = 200) perceived a shortage of nurses as a challenge. HCUs (44.94%) reported a waiting time of more than an hour at health facilities that were manned by only one or two nurses. The nurses (*n* = 86; *f* = 54; 60%) reported a work overload at the health facilities where there were fewer than two nurses, contributing to the long waiting time as described by the HCUs (*n* = 445; *f* = 245; 55%). It was interesting to note that only 23.25% of the 90 nurse respondents were trained midwives, but each nurse was expected to provide antenatal, intrapartum and postpartum midwifery care (Table 2). The shortage of nurses affected follow-up on defaulters, as indicated by 72.22% of nurses, thus negatively affecting the treatment outcome of tuberculosis and other chronic diseases. This shortage of health workers at the rural health facilities contributes to HCUs (*n =* 445; *f =* 140; 31.53%) having to travel long distances to district hospitals, which has a severe impact on their meagre financial resources.

**TABLE 2:** Midwife-trained professional nurses (professional nurses: *N* = 86).

Type of nurse (*N* = 86)	Trained as midwives				Total
	Yes		No		
	*n*	%	*n*	%	
Nurse (RGN)	5	23.80	16	76.20	21
Nurse (SCN)	7	70.00	3	30.00	10
Nurse (PHC)	8	14.80	46	85.20	54

**Total**	**20**	**23.25**	**66**	**76.74**	**86**

RGN, State Registered Nurse; SCN, State Certified Nurse; PHC, Primary Health Care.

#### Financial resources

Both nurses (*n* = 89; *f* = 79; 88.76%) and HCUs (*n* = 445; *f* = 339; 76.17%) identified a lack of financial resources as challenging. The HCU respondents (*n* =445; *f* = 397; 89.21%) indicated that 89.21% of HCUs living in rural areas do not have medical insurance to cover their healthcare needs. The nurses (*n* = 90; *f* = 86; 95.55%) confirmed this challenge and reported that HCUs try to seek free healthcare at mission health facilities. HCUs (*n* = 445; *f* = 339; 76.17%) indicated that they cannot afford to pay HCU fees at the rural health facilities or district hospitals. They then have to refrain from seeking healthcare (*n* = 445; *f* = 149; 33.48% of HCUs), or consult traditional healers (*n* = 445; *f* = 200; 44.94% of HCUs) whom they can afford. The nurses (75.55%) considered the limited financial resources amongst HCUs in rural areas and the limited access to the allocated annual fiscal funds for rural health facilities as a challenge; the nurses (75.55%) reported delayed disbursements in most cases. The lack of financial resources available to healthcare services is reflected in the annual budget allocation, where less than 10% of the total annual budget^12^ was allocated to healthcare in Zimbabwe in 2016, instead of the recommended 15% of total annual budget allocation.^12^

### Limitations and implications of the study

Because of limited financial resources, the study was conducted at the rural public health facilities, excluding district hospitals where information from health facilities is consolidated including first-line supervision. This might have contributed to limited triangulation of data. The limitations were minimised by the participation of the national health directors in the second phase of the study, where the strategic action plan was developed to enhance accessibility to healthcare in Zimbabwe.

## Discussion

**FIGURE 3:**
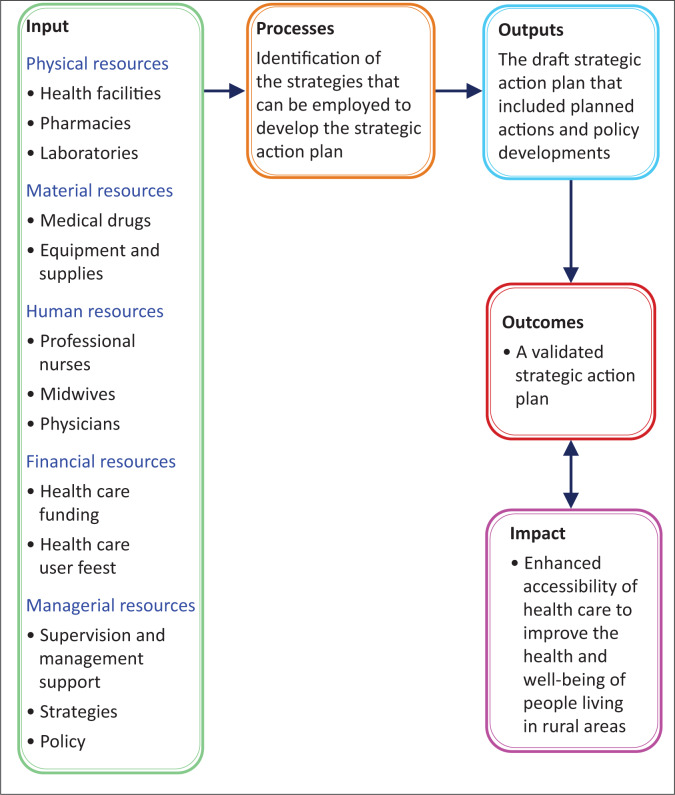
Systems Model (Forester 2009:6) as applied in the development of a validated strategic action plan to enhance accessibility to healthcare in rural areas.

The Systems Model (Figure 3),^22^ as the theoretical framework for this study, is made up of sub-systems that are interconnected and interdependent, forming a holistic healthcare system.^23^ The connected parts or components are designated as inputs, processes, outputs and outcomes. The questions were based on the inputs as described by the Systems Model that include the physical materials and human or financial resources needed to produce the outputs. Processes refer to the actions needed to change an input to contribute to an output. The outcomes refer to the association between the processes and the outputs.^23^ The accessibility of healthcare services therefore depends on the availability of physical materials, and human as well as financial resources, and the processes followed to ensure access to healthcare.^24^

How professional nurses and HCUs perceive access to healthcare in Zimbabwe was described.^25^ Some nurses (53 out of 90 [58.88%; *f* = 53]) and HCUs (226 out of 445 [50.78%; *f* = 26]) identified the lack of health facilities (within a 5-km walking distance), lack of transport and the conditions of the roads in rural areas as challenges. The distances that HCUs have to travel to access healthcare affect patient outcomes, and for this reason a maximum walking distance of 5 km to a health facility is recommended by the World Health Organization (WHO), and a distance of 8 km is recommended by the Ministry of Health and Child Care in Zimbabwe.^14,26^ The closer the health facility is to where people live, the less likely the distance will negatively influence healthcare access and healthcare-seeking behaviour. The distance that has to be travelled to access to a facility contributed to HCUs (44.94%) seeking healthcare from traditional healers who were within close proximity.^27,28^ Government is the important stakeholder that has to ensure that the distance to health facilities does not hinder accessibility or cause delays for patients seeking healthcare.^28^ The accessibility of healthcare decreases disease progression and reduces the need for hospitalisation. Therefore, developing new health facilities closer to the people can play a pivotal role in enhancing accessibility to healthcare – an input which is important according to the Systems Model.^29^ In Vietnam^30^ and China,^31^ reducing the distance to health facilities resulted in a reduction in the number of women seeking healthcare from traditional healers.

Poor road conditions between rural villages and health facilities were indicated by respondents as an obstacle when seeking healthcare. More resources to address the infrastructure shortfalls are needed, to upgrade the existing roads or construct new roads to bridge the distance between health facilities and people.^32^

Nurses and HCUs perceived access to material resources at rural health facilities as challenging, despite these being essential inputs (Systems Model) needed to enhance access to healthcare. Material resources like medicines or drugs, medical accessories such as bandages, surgical blades and cotton wool, and medical equipment like microscopes are needed for the comprehensive provision of healthcare at health facilities.^33^ The unavailability of these materials compromises the effective delivery of healthcare, as nurses (46.7%) cannot provide the healthcare and treatment needed by HCUs (59.95%). This then inevitably increases the disease burden in communities. The shortage or inconsistent supply of medical drugs at rural healthcare facilities also seriously affects patients suffering from chronic diseases like hypertension, diabetes, heart diseases and HIV. This contributes to a high incidence of and relapse in diseases such as tuberculosis in Zimbabwe, where the incidence rate was 208 per 100 000 in 2016 against the SDG target of 3.3, to end the epidemic of tuberculosis by 2030.^13^

Zimbabwe’s Health Assessment in 2010 cited in Chirwa^17^ and a study on healthcare delivery in Zimbabwe^32^ reported the same findings as the current study, indicating that only 20% of rural health facilities had essential medical drugs for the treatment of common chronic diseases. The provision of medical drugs at rural facilities is crucial to improve the health outcomes of communities.^35^

When improving the provision of medical drugs, the government should also look at addressing the shortage of health workers. Health workers are an essential part of the healthcare inputs (Systems Model) required to enhance access to healthcare.^36^ Both the nurses and HCUs in this study reported access to health workers as the greatest challenge they face. Of concern were the rural health facilities that were manned by only one or two nurses (18 out of 45 [40%]), which does not meet the minimum standard of three nurses, as determined by the Ministry of Health and Child Care’s policy.^16^ Zimbabwe has 1.25 nurses per 1000 population – far below the global median of 2.84 (three) nurses per 1000, which is indicative of a critical shortage that needs immediate resolution to enhance the accessibility of healthcare. It is particularly worrying that the government has performed so little to address these shortages, which make it impossible to provide adequate healthcare to people.^3,37^ Compounding the problem, the shortage of nurses influences the availability of midwives because the same pool of nurses is trained as midwives. According to Govule et al,^35^ at least one of the professional nurses at the health facilities should be a trained midwife to provide maternal healthcare services, and this was not the case, as a mere 23% of nurses were trained in midwifery. The nurses viewed this as a problem because inadequately trained nurses at the health facilities contribute to the work overload, demotivation and stress, thus inhibiting meaningful patient care.^36^ It also contributes to a lack of adequate time to attend to patients during consultations,^36^ as indicated by the nurse respondents in this study. One-on-one nursing care is simply impossible,^37^ because of work pressure. Nurses perceived overworking as a factor contributing to inefficiency in monitoring patients and providing inadequate support. The study findings indicated that some of the rural health facilities (40%) were manned by only one or two nurses to attend to women in labour, as well as emergency cases. In Chegutu and Masvingo rural districts, the vacancy rate was 14% for nurses.^37^ Such a situation is bound to contribute to high morbidity and mortality rates.^38^

In this study, the lack of financial resources was perceived as a challenge by both nurses and HCUs and was seen as the underlying cause of the inaccessibility of health facilities, and the shortage of medical drugs and health workers. The capacity of the Zimbabwe government to fund healthcare has been eroded, leading to costs of healthcare services surpassing HCUs’ capacity, especially at rural health facilities charging user fees. These findings were similar to those of another study conducted in Zimbabwe,^33^ which showed that vacant positions for nurses at rural health facilities were frozen because of a lack of financial resources and that HCU fees were introduced despite HCUs not being able to afford to pay the fees.

The fact that the budget was altered to attend to this issue shows the study findings were acknowledged and first steps taken in the right direction by the government.

## Conclusion

Accessing healthcare in the rural areas of Zimbabwe is a challenge, as confirmed by the sample of nurses providing healthcare within rural areas, as well as the HCUs interviewed in this study. If these challenges are not addressed, Zimbabwe might again not achieve the SDGs set by the United Nations in 2015, just as it and other countries failed to achieve the Millennium Development Goals.^34^ Zimbabwe should work towards achieving the allocation of 15% of the total fiscal budget to health, if any success in achieving the SDGs is to be expected. Governments, like the Zimbabwean government, are responsible for providing adequate, quality and accessible healthcare to their entire populations and therefore need to face the challenges and act on research findings and recommendations to increase access to healthcare.

## References

[1] Azétsop J, Ochieng M The right to health, health systems development and public health policy challenges in Chad. Philos Ethics Humanit Med. 2015;10:1 10.1186/s13010-015-0023-z 25886065 PMC4336701

[2] Chandler IR, Kizito J, Taaka L, et al Aspirations for quality healthcare in Uganda: How do we get there? Hum Resour Health. 2013;11:13 10.1186/1478-4491-11-13 23521859 PMC3610284

[3] Loewenson R, Masotya M, Mhlanga G, Manangazira P Assessing progress towards equity in health Zimbabwe Training and Research Support Centre and Ministry of Health and Child Care, Harare: Zimbabwe, in the Regional Network for Equity in Health in East and Southern Africa (EQUINET) 2014.

[4] Broni AO, Aikins I, Asbeyi O, Agyemang-Duah P The contribution of transport (road) in healthcare delivery ‘a case study of Mankranso District Hospital in the Ahafo Ano South District of Ashanti Region’. Br J Market Stud. 2014;2(4):30–51.

[5] Manjengwa J, Kasirye I, Matema C Understanding poverty in Zimbabwe: A sample survey in 16 Districts. Oxford: Centre for the Study of African Economies Conference, Economic Development in Africa; 2012.

[6] Nyazema NZ The Zimbabwe crisis and the Provision of Social services. Health Educ J Developing Soc. 2010:26(2):233–261. 10.1177/0169796X1002600204

[7] Kevany S, Murima O, Singh B, et al Socio-economic status and healthcare utilization in rural Zimbabwe: Findings from project accept. J Public Health Afr. 2012;3(1):e13 10.4081/jphia.2012.e13 PMC3436598 22962629

[8] Nyakatawa GT, Madzimbamuto FD, Shumbairerwa S, Chikumba E How inadequate availability of drugs affects anaesthesia practice in a low resource setting. Int Anesth Res Soc. 2016; 123(Suppl. 3):755.

[9] Mhere F Health insurance determinants in Zimbabwe: Case of Gweru Urban. J Appl Bus Econ. 2013;14(2):63–79.

[10] Zimbabwe National Statistics Agency Zimbabwe Demographic Health Survey 2015 Final Report. Harare: ZIMSTAT; 2016.

[11] Tafuma TA, Mahachi N, Dziwa C, et al Barriers to HIV service utilisation by people living with HIV in two provinces of Zimbabwe: Results from 2016 baseline assessment. S Afr J HIV Med. 2018;19(1):a721 10.4102/sajhivmed.v19i1.721 PMC6131723 30214827

[12] UNICEF Zimbabwe Ministry of Health and child care budget brief. Harare: UNICEF Zimbabwe; 2016.

[13] World Health Organisation World health statistics, monitoring health for the SDGs, sustainable development goals. Geneva: WHO Press; 2018.

[14] Ministry of Health and Child Welfare The national health strategy for Zimbabwe 2014–2018. Equity and quality in health: A people’s right. Harare: Government Printers; 2014.

[15] Chirwa Y, Mashange W, Chandiwana P, et al Understanding health worker incentives in post-crisis settings: Policies to attract and retain health workers in rural areas in Zimbabwe since 1997, a document review. Harare, Zimbabwe: ReBUILD Consortium; 2014.

[16] Zimbabwe National Statistics Agency & ICF International Zimbabwe Demographic and Health Survey 2015: Key indicators. Rockville, MD: Zimbabwe National Statistics Agency (ZIMSTAT) and ICF International; 2015.

[17] Ministry of Health and Child Welfare Zimbabwe National integrated health facility assessment report Dec 2011 – Jan 2012. Harare: Ministry of health; 2012.

[18] Zimbabwe National Statistics Agency Census final report 2012. Harare: ZIMSTAT 2012.

[19] Godden W Sample size formulas [homepage on the Internet]. 2004 [cited 2013 Aug 01]. Available from: http://williamgodden.com/samplesizeformula.pdf

[20] Johnson B, Christensen L 2012 Educational research: Quantitative, qualitative, and mixed approaches. 4th ed. Thousand Oaks, CA: Sage.

[21] Goldsack JC, Reilly C, Bush C, et al Impact of shortages of injectable oncology drugs on patient care. Am J Health Syst Pharm 2014;71(1):571–578. 10.2146/ajhp130569 24644117

[22] Forester BR Whatcom creek restoration project report: 2009 Technical Report. Bellingham, WA; 2009; p. 110.

[23] Cordon CP 2013 System theories: An overview of various system theories and its application in healthcare. Am J Syst Sci. 2013;2(1):13–22. 10.5923/j.ajss.20130201.03

[24] Block MAG, Akosa AB, Chowdhury M Health systems research and infectious diseases of poverty: From margins to mainstream [homepage on the Internet]. 2012 [cited 2017 Mar 21]. Available from: www.who.int/tdr/stewardship/global_report/2012/chapitre3_web.pdf

[25] MacKinney AC, Mueller KJ, Vaughn T, Zhu X 2014. From healthcare volume to healthcare value-success strategies for rural healthcare providers. J Rural Health. 2014;30(2):221–225. 10.1111/jrh.12047 24112136

[26] Patel R, Ladusingh L Do physical proximity and availability of adequate infrastructure at public health facility increase institutional delivery? A three level hierarchical model approach. PLoS One. 2015;10(12):e0144352 10.1371/journal.pone.0144352 26689199 PMC4686327

[27] Goodwin K, Tobler L Improving rural health: State policy options Health Res Serv Admin. Washington DC; National Conference of State Legislature; 2013.

[28] Boerma T, Evans D, Evans T, et al World Health Organisation and World Bank Tracking universal health coverage, first global monitoring report. Geneva; 2015.

[29] Kadobera D, Sartorius B, Masanja H, Mathew A, Waiswa P 2012. The effect of distance to formal health facility on childhood mortality in rural Tanzania. Glob Health Action. 2012;5:19099 10.3402/gha.v5i0.19099 PMC3495250 23151364

[30] Tran BX, Nguyen LH, Nong VM, Nguye CT Health status and health service utilization in remote and mountainous areas in Vietnam. Health Qual Life Outcome. 2016;14:85 10.1186/s12955-016-0485-8 PMC4895985 27267367

[31] Hu R, Dong S, Zhao Y, Hu H, Li Z Assessing potential spatial accessibility of health services in rural China: A case study of Donghai County. Int J Equity Health [serial online]. 2013 [cited 2014 May 20]. 12:35 Available from: http://www.equityhealthj.com/content/12/1/35 10.1186/1475-9276-12-35 PMC3747861 23688278

[32] MacKinnon J, MacLaren B Human resources for health challenges in fragile states: Evidence from Sierra Leone, South Sudan and Zimbabwe. Ontario, Canada: The North-South Institute; 2012.

[33] Grignon JS, Ledikwe JH, Makati D, Nyangah R, Sento BW, Semo B 2014. Maximizing the benefit of health workforce secondment in Botswana: An approach for strengthening health systems in resource-limited settings. Risk Manage Healthc Pol. 2014;7:91–98. 10.2147/RMHP.S61473 PMC4036141 24876798

[34] Smith D, Roche E, O’Loughlin K, et al 2015. Satisfaction with services following voluntary and involuntary admission. J Ment Health. 2015;23(1):38–45. 10.3109/09638237.2013.841864 24484191

[35] Govule P, Mugisha JF, Katongole SP, et al Application of Workload Indicators of Staffing Needs (WISN) in determining health workers’ requirements for Mityana General Hospital, Uganda. Int J Publ Health Res. 2015;3(5):254–263.

[36] Nkwo PO, Lawani LO, Ezugwu EC, Iyoke CA, Ubesie AC, Onoh RC Correlates of poor perinatal outcomes in non-hospital births in the context of weak health system: The Nigerian experience. BMC Pregnancy Childbirth. 2014;14:341 10.1186/1471-2393-14-341 25271134 PMC4262084

[37] Zimbabwe Health Services Board 2015 Report on the state of human resources for health in Zimbabwe. Harare: Nkala B and Gotora B; 2015.

[38] Asante A, Price J, Hayen A, Jan S, Wiseman V Equity in healthcare financing in low- and middle-income countries: A systematic review of evidence from studies using benefit and financing incidence analyses. PLoS One. 2016;11(4):e0152866 10.1371/journal.pone.0152866 27064991 PMC4827871

